# Rapid and cumulative adult plasticity in the mouse visual cortex

**DOI:** 10.3389/fncir.2025.1537305

**Published:** 2025-02-28

**Authors:** Hiroyuki Miyamoto, Emi Mazaki, Yuichi Makino, Qi Fang, Tomohito Hamada, Youichi Handa, Takao K. Hensch

**Affiliations:** ^1^International Research Center for Neurointelligence (IRCN), The University of Tokyo Institutes for Advanced Study, Tokyo, Japan; ^2^Technology and Innovation Center, Daikin Industries, Ltd., Osaka, Japan; ^3^Department of Molecular Cellular Biology, Center for Brain Science, Harvard University, Cambridge, MA, United States; ^4^FM Kirby Neurobiology Center, Boston Children's Hospital, Harvard Medical School, Boston, MA, United States

**Keywords:** experience-dependent adult plasticity, mouse visual cortex, flash-evoked potentials, NMDA receptors, NREM sleep, stimulus-selective response plasticity (SRP)

## Abstract

Experience-dependent neural plasticity enables the brain to adapt to diverse and dynamic environments by reshaping circuits. In the adult visual system, this plasticity can be elicited by repeated sensory stimuli; however, its temporal dynamics and underlying mechanisms remain unclear. Here, we investigated the regulation of visual response potentiation induced by repeated light flashes in the primary visual cortex of awake adult mice. Our findings revealed two distinct temporal phases of potentiation: a rapid phase occurring within seconds and a cumulative phase developing over hours to days. Notably, the identification of this rapid phase phenomenon adds to and refines the prevailing view that visual plasticity in the adult cortex is predominantly slow. Additionally, exposure to visual stimuli enhanced spontaneous slow-wave activity in the visual cortex during non-REM sleep. This plasticity was significantly impaired in Grin2a (NR2A) knockout mice, a model of schizophrenia, which mirrors visual plasticity deficits observed in human patients. The dual temporal characteristics of flash-evoked visual plasticity likely reflect multifaceted aspects of adult brain functionality, encompassing processes related to memory, learning, and neurological disorders. This model of visual plasticity in defined neural circuits provides a simplified yet robust and extensible framework for exploring the neural mechanisms underlying adaptive and maladaptive behavioral changes.

## Introduction

Neuronal plasticity underpins the brain’s capacity to develop, adapt through learning and memory, and is involved in various neurological disorders (O'Reilly et al., 2019; Magee and Grienberger, 2020; Fernandez and Luthi, 2020). Visual system plasticity serves as a framework for understanding fundamental cognitive processes, such as information filtering, processing, representation, storage, and retrieval, which are critical for learning and memory. The primary visual cortex (V1), with its well-characterized anatomy and physiology, has historically provided a unique model to elucidate the molecular, circuit-level, and physiological mechanisms underlying neural plasticity. Leveraging these advantages, this study addresses key gaps in the current understanding of adult visual plasticity.

One of the most prominent models of cortical plasticity is ocular dominance plasticity, wherein visual deprivation during development induces long-lasting changes in cortical responsiveness (Hensch, 2005). Ocular dominance plasticity has been regarded as a minimal model for the relationship between critical periods and brain development. Notably, this has led to remarkable advancements in molecular and cellular-level insights into synaptic plasticity *in vivo*, further elucidating molecular strategies for reactivating critical period plasticity (Hensch and Quinlan, 2018). However, its expression is typically confined to developmental windows, making chronic and stable recordings in unanesthetized juvenile mice challenging. Moreover, the inherent weakening of responses from the deprived eye complicates longitudinal analyses of visual circuits in the same individual.

Although historically associated with early development, recent research has demonstrated that the adult cortex also exhibits visual experience-dependent plasticity. For example, repeated exposure to gratings of a particular orientation selectively enhances visual responses to the same orientation in head-fixed, awake mice, assessed via visually evoked potentials (VEPs) (Frenkel et al., 2006; Montgomery et al., 2022) and neuronal activity (Aton et al., 2014; Cooke et al., 2015; Makino and Komiyama, 2015; Durkin and Aton, 2016; Kaneko et al., 2017; Lantz and Quinlan, 2021). This enhancement, known as stimulus-selective response plasticity (SRP), likely contributes to visual perceptual learning (Cooke et al., 2015; Montgomery et al., 2022), sequential learning and behavioral habituation, a form of long-term visual recognition memory (Cooke et al., 2015; Gavornik and Bear, 2014). A series of studies conducted by Bear and colleagues (Frenkel et al., 2006; Montgomery et al., 2022) revealed that the early components of VEPs (< 200 msec post-stimulus onset) exhibit significant enhancement. These responses are orientation-selective, occur within V1 without ocular transfer, and demonstrate NMDA receptor-dependence (Frenkel et al., 2006). Parvalbumin-positive inhibitory interneurons are implicated in responses to novel orientations, while somatostatin-positive inhibitory interneurons mediate habituation (Kaplan et al., 2016; Hayden et al., 2021). Distinct plasticity processes in the soma and dendrites associated with SRP have also been reported (Kim et al., 2020). Recent evidence further highlights the role of intracortical circuits, not only thalamocortical feedforward input, in these processes (Hayden et al., 2023). Importantly, SRP emerges hours or days after visual exposure (Frenkel et al., 2006) and is facilitated by non-REM (NREM) sleep following stimulation (Durkin and Aton, 2016; Durkin et al., 2017). SRP impairments have also been reported in a mouse model of neurodegenerative disease (Papanikolaou et al., 2022).

In the early visual cortex, orientation selectivity, which is critical for contour detection, arises from the spatial alignment of thalamic inputs to cortical neurons. Visual responses to orientation gratings (pattern VEPs) are commonly used to assess visual function in humans and animals (Porciatti et al., 1999; Valstad et al., 2021). Flash VEPs, triggered by brief spatially unstructured light flashes, offer an alternative measure. Although both pattern and flash VEPs share a common current flow pathway from layer 4 to the infragranular and supragranular layers, their distinct latencies and amplitudes suggest different underlying mechanisms (Schroeder et al., 1998). Rapid increases in VEP amplitude in response to repetitive flash stimuli have been reported in both rats (Dyer, 1989; Herr et al., 1991; Herr et al., 1994) and mice (Uhlrich et al., 2005). This simple and practical method complements SRP as a model for studying adult visual plasticity. However, detailed similarities and differences between flash VEPs and SRP remain unclear. Additionally, variability in visual responses due to animal behavior and postural changes poses challenges for accurate quantification in freely moving animals. Thus, the detailed time course and quantification of visual plasticity remains to be determined. Even a short duration of visual stimulation (less than 1 msec) induces enduring effects (approximately 0.6 s) in the visual cortex (Padnick and Linsenmeier, 1999) (this study). Consequently, high-frequency stimulation (8 Hz) (Uhlrich et al., 2005) may cause temporally merged responses, making it difficult to isolate VEP peaks.

Flashing light stimulation offers a straightforward and effective method for examining visual function. With brief stimulus durations (10 μsec to 10 msec) and low-frequency repetition (≤ 1 Hz), flash stimulation minimizes complexity and potential confounds in visual processing. Brief flashes, which resemble the light and dark transitions commonly encountered in natural environments, can be considered as a type of visual stimulus that complements the orientation-selective stimuli typically used in SRP experiments, offering insights into naturalistic visual processing. Furthermore, flash stimulation allows simultaneous monitoring of responses from both ipsilateral and contralateral eye inputs (Griffen et al., 2017). In this study, we explored visual plasticity induced by repetitive flashing light (10 msec at 0.5 Hz) in awake, head-fixed mice by controlling light direction and distance. We observed that flash-evoked visual plasticity (FVP) followed two distinct temporal phases: rapid potentiation within seconds to minutes and gradual potentiation over hours to days. These temporally distinct phases of FVP, differing from SRP, may provide a simplified model for studying experience-dependent circuit reorganization relevant to learning, development, and neuropsychiatric disorders.

## Methods

### Animals

All animal care and use procedures were approved by the Animal Experiment Committee of the University of Tokyo. Adult C57BL/6 J mice (Japan SLC, both sexes, 3–6 months old) were housed under controlled conditions (23°C, 55% humidity) on a 12-h light/12-h dark cycle (lights on at 8:00 and off at 20:00). Food and water were available ad libitum, with fewer than five animals per cage. Grin2a (NR2A) homozygous knockout mice were backcrossed to a C57BL/6 J background for over 11 generations using breeding pairs originally provided by Dr. M. Mishina (University of Tokyo) (Fagiolini et al., 2003).

### Electroencephalogram (EEG), electromyogram (EMG), and local field potential (LFP) recordings in head-fixed mice

Under 1–1.5% isoflurane anesthesia (25–30 g body weight at surgery), stainless steel screws (1.1 mm diameter) were implanted over the right somatosensory cortex (1.5 mm lateral to the midline, 1.0 mm posterior to bregma) for EEG recordings. A reference electrode was implanted in the cerebellum (midline, 1.5 mm posterior to lambda). For EMG recordings, a stainless steel wire (100 μm) was inserted into the cervical trapezius muscle. For monopolar LFP recordings, insulated stainless steel wires (200 μm diameter) were stereotaxically implanted to target the anterior cingulate cortex (0.6, 0.3, 1.2), V1 binocular zone (medial: −3.4, 2.6, 0.4; lateral: −3.4, 3.0, 0.4), V2 higher-order cortical visual area lateromedial (V2LM: −3.4, 3.6, 0.4) (Wang and Burkhalter, 2007), and the corresponding regions in the left hemisphere. LFP electrodes were implanted 400 μm below the cortical surface in the binocular zone of V1, primarily targeting layer 4 and the upper portion of layer 5. These layers are known to produce the largest VEP responses in the SRP experiments (Frenkel et al., 2006). Electrodes were secured with petroleum jelly and dental acrylic. An aluminum frame (CF-10; Narishige, Tokyo, Japan) was attached to dental acrylic for head fixation. Local anesthesia (lidocaine) and antibiotics (ampicillin) were administered during surgery, and buprenorphine (0.05 mg/kg) was subcutaneously administrated for postoperative analgesia. After a one-week recovery period, animals were gradually habituated to the recording chamber and head-fixation setup until they remained calm for at least 30 min.

### Visual stimulation

**Figure 1:**
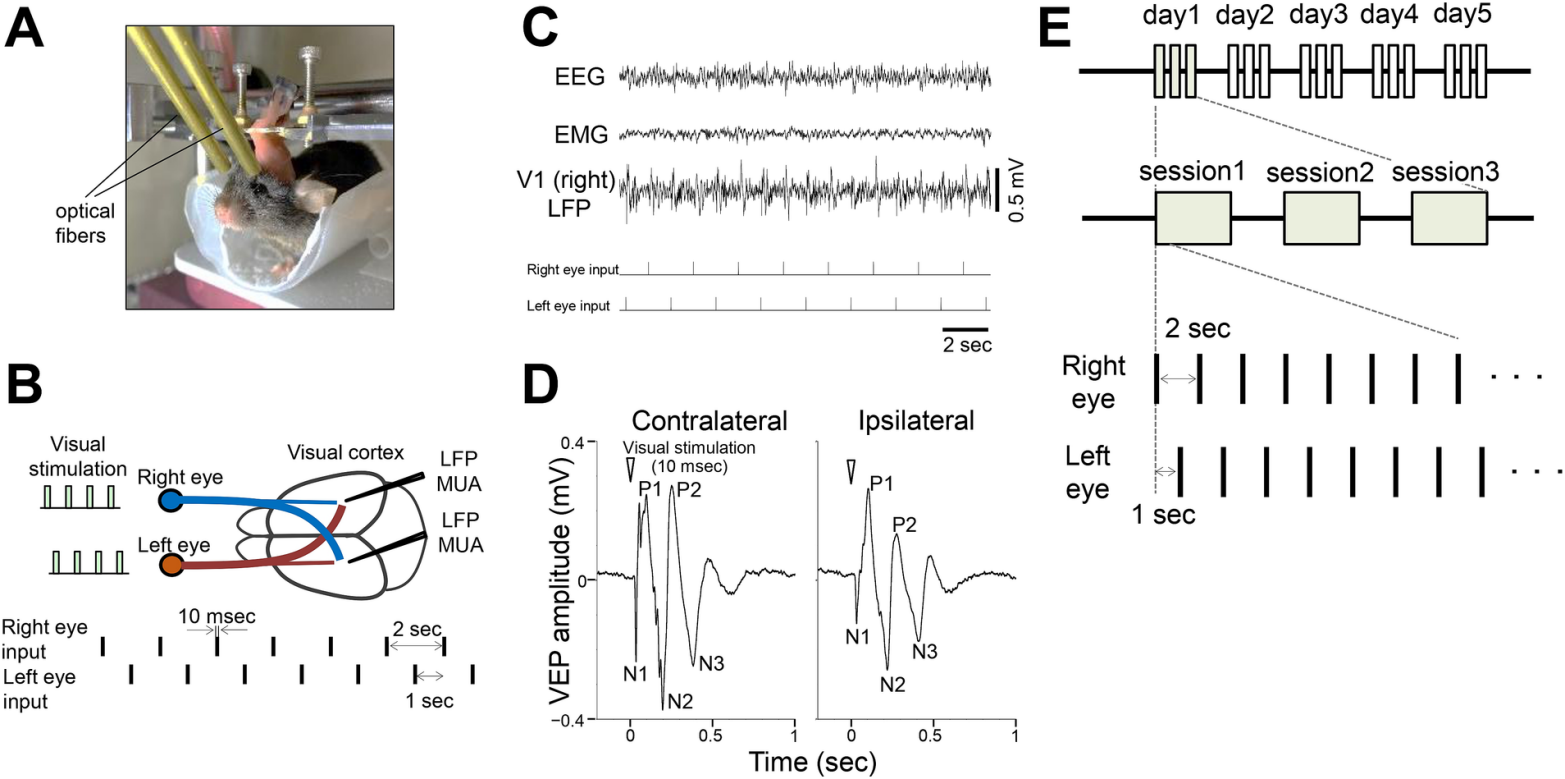
Flashing light visual stimulation of awake mice **(A)** Experimental setup for visual stimulation in a head-fixed awake mouse. Optical fibers connected to LEDs were positioned in front of each eye. **(B)** Local field potentials (LFPs) and multiunit activity (MUA) were recorded from the binocular zone of the primary visual cortex (V1). Flashing light stimuli (10 msec duration) were alternately delivered to the right or left eye at 1 s intervals. **(C)** Representative traces showing somatosensory EEG, cervical EMG, and V1 LFP (right hemisphere) during visual stimulation (ticks indicate the stimulation events). Stimulation of either eye evoked visual responses in both ipsilateral and contralateral visual cortices. **(D)** Representative visually evoked potential (VEP) in V1 (averaged LFP responses to 300 stimulations per eye). N1, N2, and N3 represent the first, second, and third negative peaks, respectively, while P1 and P2 represent the first and second positive peaks, respectively. **(E)** Timeline of the visual experiments. Visual stimulation sessions consisted of alternating presentations to the left and right eyes, with 100 presentations per eye for a total of 200 presentations (200 s). Each day included 3 sessions, conducted over at least 4 days.

Flash stimulation was delivered using two fiber-coupled white LEDs (1.3 mm diameter) with optical fibers directed at each eye to cover the binocular zone (Figure 1A). The timing and duration of LED flashes were controlled by a TTL pulse generator (OPTG8, Doric Lenses, Quebec, Canada) and a constant-current stimulator (SEG-3104, Nihon Kohden, Tokyo, Japan). Light intensity was maintained at 100 cd/m^2^ throughout the experiment. Visual stimulation began after an 8 min dark adaptation, with alternating 1 Hz flashes (10 msec duration) to each eye. Each eye received 100 flashes per session, repeated three times (300 flashes total) with 300 s intervals between repetitions. Sessions were conducted daily for 4–10 days (mean = 6.8 days). EEG, EMG, LFP, and LED timing data were recorded simultaneously and animal behavior was monitored using an infrared camera. Variations in the protocol are described in the respective sections.

### Multiunit activity (MUA) recording

Extracellular spike activity was recorded using bipolar electrodes made of twisted stainless wires (100 μm), implanted bilaterally in the binocular zone of V1 (−3.4, ±3.0, 0.4). A reference electrode with minimal neuronal activity was selected. Signals were band-pass filtered (0.15–8 kHz) and digitized at 40 kHz (MAP system, Plexon, Dallas, TX) whenever the signal exceeded a preset threshold of 4 standard deviations above the baseline. LFPs were recorded simultaneously with MUA. Electrode positions were marked post-experiment by electrolytic lesioning (50 μA for 10 s) and confirmed histologically. MUA data were processed using Offline Sorter (Plexon), and LFP and MUA analyses, including spike-triggered averages, were performed using NeuroExplorer (Nex Technologies, Madison, WI).

### Chronic recordings in behaving mice

For chronic recordings, tethered mice with implanted electrodes were connected to a 16-channel commutator (Plexon) and allowed to move freely within a shielded cage. LFPs were recorded (filtered 0.7–170 Hz, sampled at 1 kHz) using the MAP system and analyzed offline. Visual stimuli (10 msec duration, 1 Hz, 3 × 100 flashes with 400 s intervals) were presented over 4 days using an LED array positioned above the cage (30–60 lx at floor level). Animals were kept awake by gentle tapping on the cage during visual stimulation in the dark. Sleep recordings were performed for 7–10 days to assess the relationship between visual plasticity and sleep. Sleep stages were scored manually based on EEG and EMG signals.

### Drug administration

The NMDA receptor antagonist MK801 (FUJIFILM Wako Pure Chemical Corporation, Osaka, Japan) was dissolved in saline (0.1 mg/mL) and administered intraperitoneally (0.5 mg/kg) 15 min before daily recording sessions for 6 consecutive days. For ACC inactivation, the GABA_A_ receptor agonist muscimol (Merck, Darmstadt, Germany) was dissolved in saline (1 or 10 mM) and applied unilaterally (0.3 μL) to the ACC via a microsyringe 30 min before recording under light isoflurane anesthesia. The injection cannula remained in place for 30 s post-injection. A guide cannula (C313G, 7 mm length, P1 Technologies, Roanoke, VA) and an internal dummy cannula (C313DC, 8 mm length) were implanted along with the EEG/EMG and LFP recording setups. The injection cannula tip (C313I, 360 μm diameter, 8 mm length) was extruded 1 mm from the guide cannula end, targeting the ACC (0.6, 0.3, 1.2) ipsilateral to the EEG recording site.

### Support vector machine (SVM) analysis

SVM training and classification were performed with minor modifications to previously described methods (Makino et al., 2019). Positive and negative peak amplitudes were extracted from each VEP event by detecting the maximum and minimum potentials within a specified time window. Additionally, the total area under the VEP waveform was calculated by summing all areas above and below the baseline between 0 and 500 msec after visual stimulation. One or more of these indices, derived from one or more electrodes, were used for SVM training and classification. Randomly selected 75% of VEP events were used as the training dataset, while the remaining 25% were classified as left or right eye stimulation events using the fitcsvm and predict functions in MATLAB (MathWorks, Natick, MA) with a linear kernel. This process was repeated 1,000 times with different datasets, and mean classification accuracy was calculated for each mouse.

### Statistics

Data are presented as mean ± SEM. Statistical analyses were performed using Prism 9 (GraphPad Software, La Jolla, CA). Two-sided unpaired Student’s *t*-tests were used for comparisons between control and experimental groups, unless otherwise stated. For data with significantly different variances, Mann–Whitney U tests or Welch’s corrected *t*-tests were applied. Statistical significance was set at *p* < 0.05.

## Results

### Responses to flashing light stimulation in the visual cortex of awake mice

We investigated visual responses in the primary visual cortex (V1) of awake head-fixed adult mice (3–6 months old) during brief light stimulation targeting the binocular zone. Optical fibers connected to a white LED were securely positioned near the eyes and remained fixed throughout the experiments (Figure 1A). Neurons in the binocular zone of V1 receive inputs from both eyes, allowing quantification of responses to ipsilateral and contralateral stimuli (Figure 1B). Each eye was stimulated sequentially with brief flashes (10 msec) at a low frequency (0.5 Hz) with a 1 s interval between the eyes. Local field potential (LFP) recordings in V1 showed responses to visual stimulation from each eye (Figure 1C). Stimulus-triggered averaged visual responses, visually evoked potentials (VEPs), revealed distinct characteristic components: an early negative peak (N1, ~30 msec latency), a first positive peak (P1, ~100 msec), a second negative peak (N2, ~200 msec), a second positive peak (P2, ~300 msec), and a third negative peak (N3, ~400 msec), lasting approximately 600 msec (Figure 1D). N1 likely reflects input from the lateral geniculate nucleus, while later components may involve intracortical processing (Schroeder et al., 1998). Using this setup, we performed repeated light stimulation experiments for at least 4 days, with 3 sessions per day (Figure 1E).

### Potentiated visual responses following repeated stimulation

**Figure 2:**
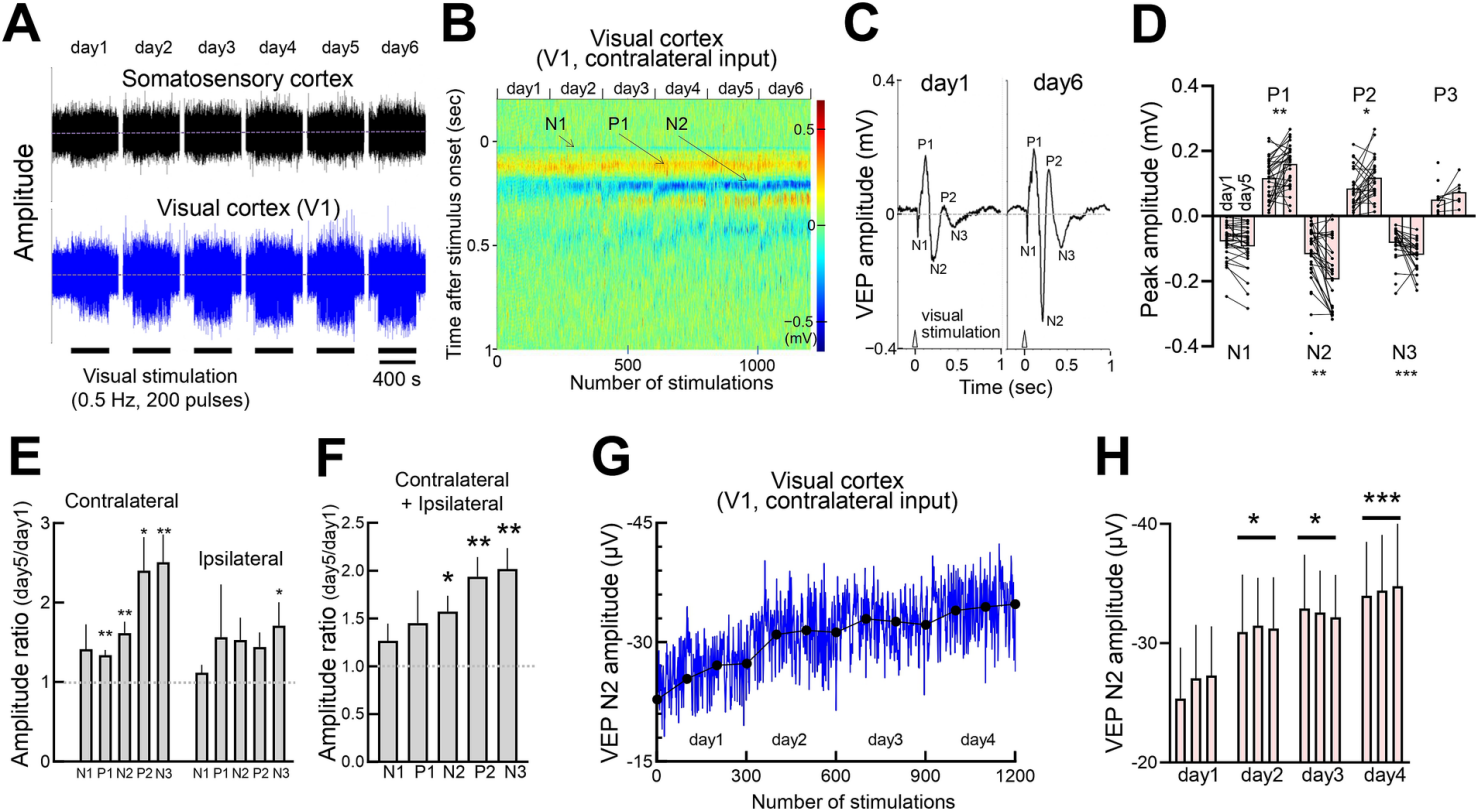
Potentiation of response amplitudes induced by repetitive visual stimulation **(A)** Concurrent recordings of somatosensory EEG and visual cortex LFP over 6 consecutive days. Each day, 200 visual stimulations (10 msec light flashes) were provided to each eye (400 stimulations in total). **(B)** Time course of color-scaled individual visual responses in V1, showing responses to 200 flashes per day over 6 days (total 1,200 flashes). Yellow and blue indicate the positive and negative VEP peaks, respectively. **(C)** Representative VEPs evoked by flashing light in V1 on day 1 and day 6. **(D)** Comparison of VEP peak amplitudes on days 1 and 5 across 8 mice, showing responses in the right and left binocular cortices to contralateral and ipsilateral inputs. Mann–Whitney test, N1:15 recording sites, 30 recordings of contralateral or ipsilateral eye stimulation, *p* = 0.4001; P1:15 recording sites, 30 recordings, **p = 0.0080; N2:15 recording sites, 30 recordings, **p = 0.0089; P2:14 recording sites, 27 and 28 recordings, *p = 0.0450; N3:14 recording sites, 27 and 26 recordings, ***p = 0.0007; P3:6 recording sites, 12 and 7 recording sites, *p* = 0.1956. *p < 0.05, **p < 0.01, ***p < 0.001, ****p < 0.0001. **(E)** Potentiation of VEP peak amplitudes following contralateral or ipsilateral eye visual stimulation. The ratios of day 5 to day 1 VEP peaks (1.0: no change) are plotted. Visual responses of the right and left cortices were averaged for each mouse. *N* = 8 mice, 7 and 8 recording sites. One-sample *t*-test, theoretical mean = 1. Contralateral responses, N1: *p* = 0.2259, *t* = 1.328, degree of freedom (df) = 7; P1: **p = 0.0013, *t* = 5.168, df = 7; N2: *p = 0.0035, *t* = 4.305, df = 7; P2: *p = 0.0162, *t* = 3.310, df = 6; N3: **p = 0.0049, *t* = 4.340, df = 6. Ipsilateral responses, N1: *p* = 0.2590, *t* = 1.228, df = 7; P1: *p* = 0.4229, *t* = 0.8510, df = 7; N2: *p* = 0.1014, *t* = 1.885, df = 7; P2: *p* = 0.0536, *t* = 2.396, df = 6; N3: *p = 0.0484, *t* = 2.386, df = 7. **(F)** Pooled VEP data (contralateral and ipsilateral responses are averaged for each mouse). In each animal, the VEP peak values were calculated as follows: the ipsilateral responses in the right-hemispheric visual cortex to right-eye stimulation and in the left-hemispheric visual cortex to left-eye stimulation were averaged for each mouse, as were the contralateral responses under both conditions. The individual averages for ipsilateral and contralateral responses were then averaged across all 8 animals. One-sample *t*-test, N1:*p* = 0.1769, *t* = 1.501, df = 7; P1: *p* = 0.2272, *t* = 1.324, df = 7; N2: *p = 0.0104, *t* = 3.472, df = 7; P2: **p = 0.0026, *t* = 4.567, df = 7; N3: **p = 0.0024, *t* = 4.626, df = 7. **(G)** Time course of VEP N2 amplitude (contralateral VEP in the left V1, blue line) in another set of animals (6 mice averaged) with daily visual stimulation (3 sets of 100 light pulses interleaved for 400 s) for 4–6 consecutive days. The average VEP amplitudes for each 100 pulses session are indicated by filled circles. **(H)** Average N2 amplitudes (every 100 stimulations of the contralateral input). *N* = 6 mice. Statistical comparisons were performed between the first and subsequent days (average of 300 stimulations). Paired *t*-test, day 1 vs. day 2, *p = 0.0197, *t* = 3.380, df = 5; day 1 vs. day 3, *p = 0.0161, *t* = 3.565, df = 5; day 1 vs. day 4: ***p = 0.0005, *t* = 7.924, df = 5.

Repeated light stimulation led to a gradual increase in V1 LFP amplitude, while somatosensory EEG activity remained unchanged (Figure 2A). Progression from the initial to the final stimulation revealed that P1 and N2 peaks became more prominent, with P2 and N3 peaks emerging later, while N1 remained stable (Figure 2B). Comparison of VEPs between day 1 and day 6 demonstrated a pronounced increase in N2 amplitude (Figures 2C,D). This response gain was generally greater for contralateral inputs than for ipsilateral inputs (Figure 2E). Combined data from both eyes showed significant increases in N2, P2, and N3 amplitudes by day 5, while N1 remained unchanged (Figure 2F). The time course of N2 amplitude exhibited steady growth, reaching a plateau around day 4 (Figures 2G,H).

### Rapid potentiation of visual responses

**Figure 3:**
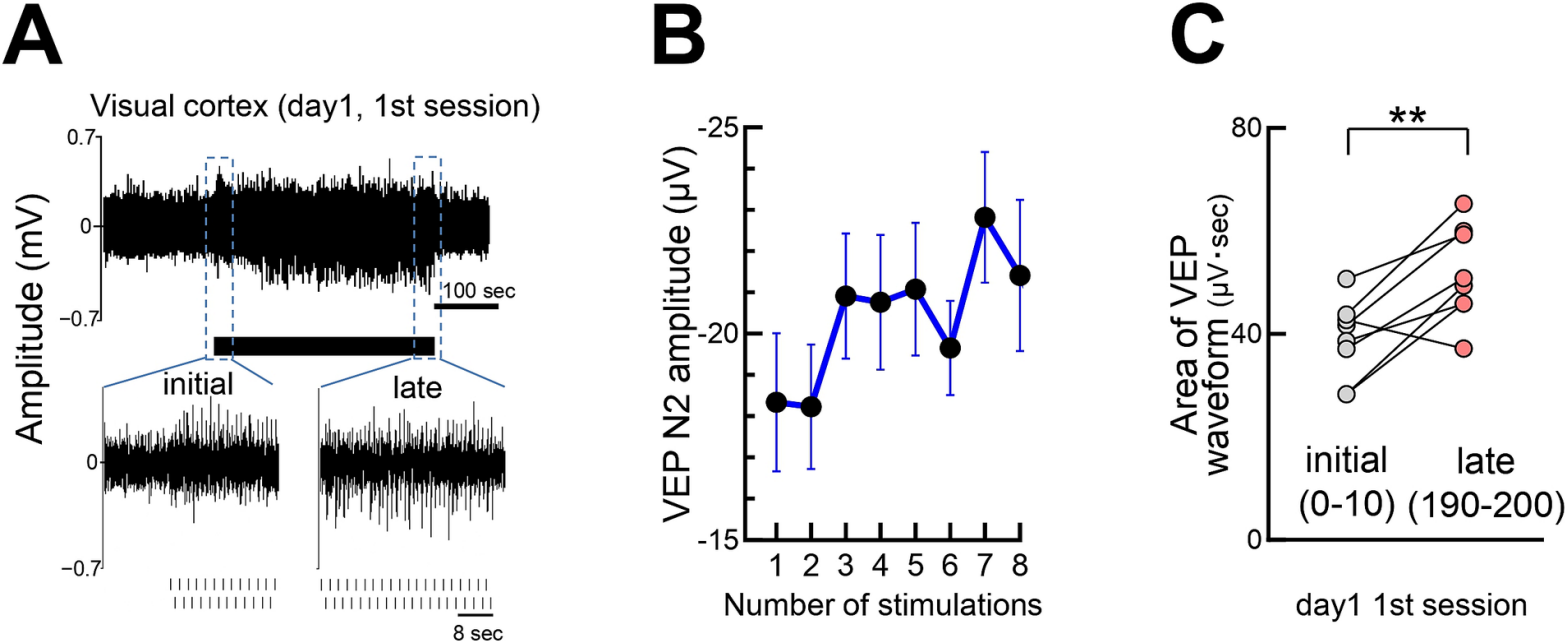
Rapid growth of visual responses during stimulation **(A)** Visual responses during the first 200 stimulations (100 per eye) on day 1, with responses for the first 25 and last 38 stimulations. **(B)** Rapid increase in the VEP N2 peak amplitude within seconds of stimulation onset. *N* = 14 mice, 1 s stimulus intervals. **(C)** A significant increase in the V1 visual response (area under waveform) from the initial (0–10 pulses) to late (190–200 pulses) stimulation on day 1. Paired *t*-test, **p = 0.0054, 8 mice, *t* = 3.971, df = 7. At the beginning of the stimulation, individual visual responses were weak; thus, the area of the waveforms was quantified instead of the N2 amplitude.

In addition to gradual potentiation, visual responses increased rapidly within seconds of stimulation onset (Figure 3A). Initially weak responses strengthened, eventually resembling a typical VEP waveforms. Quantitative analysis of response strength, comparing early (first 0–10) with later (190–200) stimulations, confirmed rapid potentiation (Figures 3B,C). The rapid potentiation of VEPs was maintained even after the termination of the visual stimulation session and persisted when the next session began on day 1 and day 2 (Supplementary Figure S1). We refer to the cumulative process observed over several days (Figure 2) and the short-term enhancement (Figure 3) collectively as flash-evoked visual plasticity (FVP). Similar FVP was observed in young mice (P30), around the critical period for ocular dominance plasticity (Supplementary Figure S2).

The observed rapid potentiation may reflect changes in brain states, such as increased alertness or attention, rather than purely visual plasticity. We examined changes in the brain state during visual stimulation. Specifically, we quantified beta activity (20–30 Hz) and gamma activity (30–100 Hz) during the first session of visual stimulation by comparing the pre-stimulation, stimulation, and post-stimulation phases. EEG beta activity in the somatosensory cortex is commonly considered a marker of alertness or wakefulness, whereas gamma activity of LFP in the anterior cingulate cortex (ACC) is often associated with attention. Our results indicate that while beta and gamma activities in the V1L region increased markedly during stimulation, the increases in the EEG (somatosensory cortex) and ACC were more gradual and transient, peaking shortly after the onset of visual stimulation (Supplementary Figure S3). These findings suggest that changes in alertness or attention may contribute to the rapid process but are unlikely to fully account for it.

### Regional specificity of FVP

**Figure 4:**
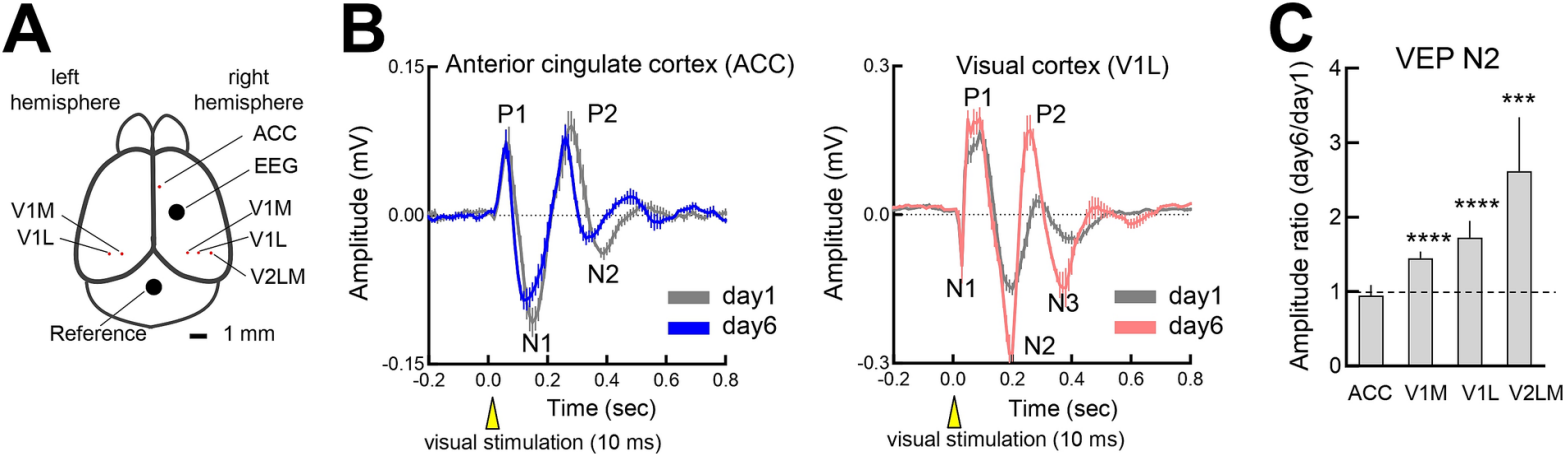
Flash-evoked visual plasticity (FVP) in visual processing regions **(A)** LFP recording sites in the somatosensory cortex, anterior cingulate cortex (ACC), V1 (medial and lateral binocular regions) and V2LM. **(B)** (Left) VEP changes from the initial session (day 1, 300 pulses, gray line) to the late session (day 6, 300 pulses, blue line) in the ACC (4 mice). (Right) VEP changes from the initial session (day 1, 300 pulses, gray line) to the late session (day 6, 300 pulses, blue line) in V1BL (6 mice). The LFPs of the ACC and V1BL were simultaneously recorded. **(C)** Day 6 to day 1 ratio of VEP N2 peaks (ipsilateral and contralateral responses) across the regions. Wilcoxon signed-rank test, *N* = 6 mice. ACC: 4 recording sites, *p* = 0.8750; V1M, 19 recording sites; ***p = 0.0001; V1L: 21 recording sites, ***p = 0.0001; V2LM: 12 recording sites, ***p = 0.0005.

To determine whether FVP varies across cortical regions, we examined V1 (medial and lateral binocular zones), V2LM, and ACC, which are involved in attention, anticipatory activity and memory processing (Makino et al., 2019; Sidorov et al., 2020), using simultaneous LFP recordings (Figure 4A). While the V1 and V2LM regions displayed robust FVP, the ACC did not show any changes after repeated visual stimulation (Figure 4B). The increase in N2 amplitude was significant in the visual areas, but not in the ACC, paralleling the lack of somatosensory EEG changes (Figure 4C). Given the long latency of the N2 peak (~200 msec), cortico-cortical or cortico-subcortical interactions may contribute to the FVP. However, muscimol-induced inhibition of ACC activity after establishing the FVP did not affect V1 responses (Supplementary Figures S4A,B), suggesting that the FVP is localized within the visual cortex.

### Eye input pathway-specific FVP

**Figure 5:**
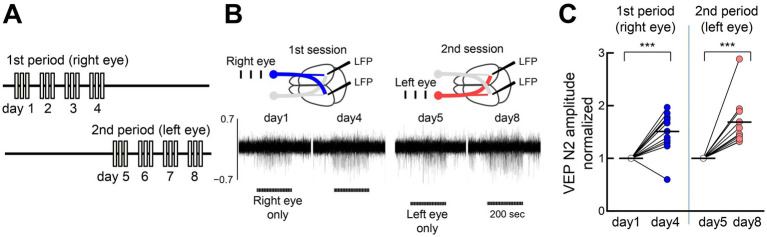
Eye input specificity for FVP **(A)** Sequential visual stimulation of one eye (left panel, 1st period, 300 pulses/day for 4–5 days) and then to the other eye (right panel, 2nd period, 300 pulses/day for 4–5 days) in the same mice. **(B)** Representative mouse data are shown. Stimulation of the right eye (blue) activates both the contralateral left visual cortex and the ipsilateral right visual cortex. After stimulating the right eye for only 4 days, the left eye was stimulated exclusively for another 4 days. Horizontal bars represent one session of visual stimulation (100 trials). **(C)** Both visual stimulation sequences induce potentiation at the same recording sites. VEP peak N2 amplitudes of 3 mice and visual responses of the right and left cortices to contralateral and ipsilateral eye inputs were included (12 samples). Paired *t*-test, 3 mice, 12 recording sites, 1st period: **p = 0.007, *t* = 4.625, df = 11; 2nd period: ****p < 0.0001, *t* = 10.06, df = 11.

Because neurons in V1’s binocular zone receive inputs from both eyes, we investigated pathway-specific FVP. We first stimulated one eye for 4–5 days, and then stimulated the other eye for 4–5 days in the same animals (Figures 5A,B). Both stimulations significantly increased the N2 amplitude (~150% increase in each session), comparable to alternating eye stimulation (Figure 5C). This finding suggests pathway-selective plasticity and a locus of initial plasticity in the V1.

### Neural activity during FVP

**Figure 6:**
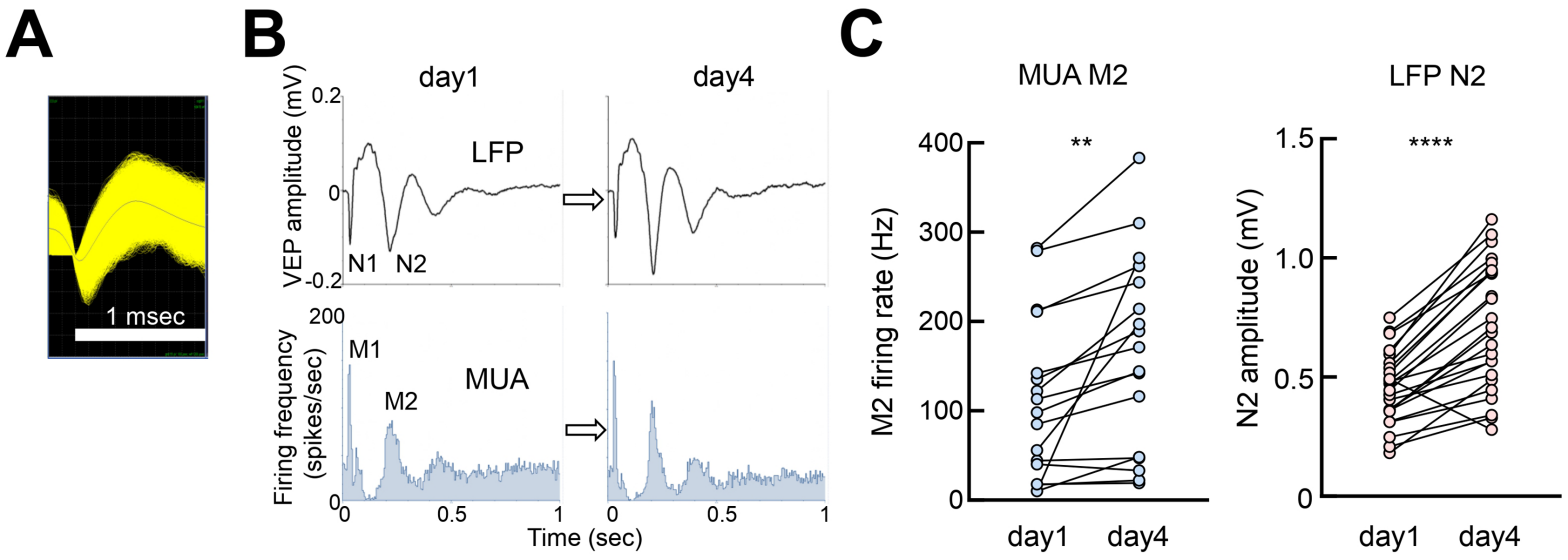
Neuronal spiking activity associated with VEP **(A)** Example of multiunit activity (MUA) from V1 (putative pyramidal neurons, 58,120 spikes overlaid). **(B)** Perievent histogram of MUA peaks M1 and M2, corresponding to VEP peaks N1 and N2 (average of 300 pulses). **(C)** Paired *t*-test, day 1 vs. day 4, MUA M2:12 recording sites (ipsilateral and contralateral responses), 17 MUAs, *t* = 3.470, df = 16, **p = 0.0032; VEP N2:24 recording sites (ipsilateral and contralateral responses), *t* = 7.685, df = 23. ****p < 0.0001.

LFP, multiunit activity (MUA) recordings from the same electrode showed a close association between VEP negative peaks and neuronal activity (Figures 6A,B). The MUA peaks M1 and M2 were aligned with the VEP peaks N1 and N2, respectively. Neuronal activity significantly increased with VEP amplitudes, indicating that changes in VEPs closely corresponded to local cortical activity (Figure 6C).

### Decoding of brain responses

**Figure 7:**
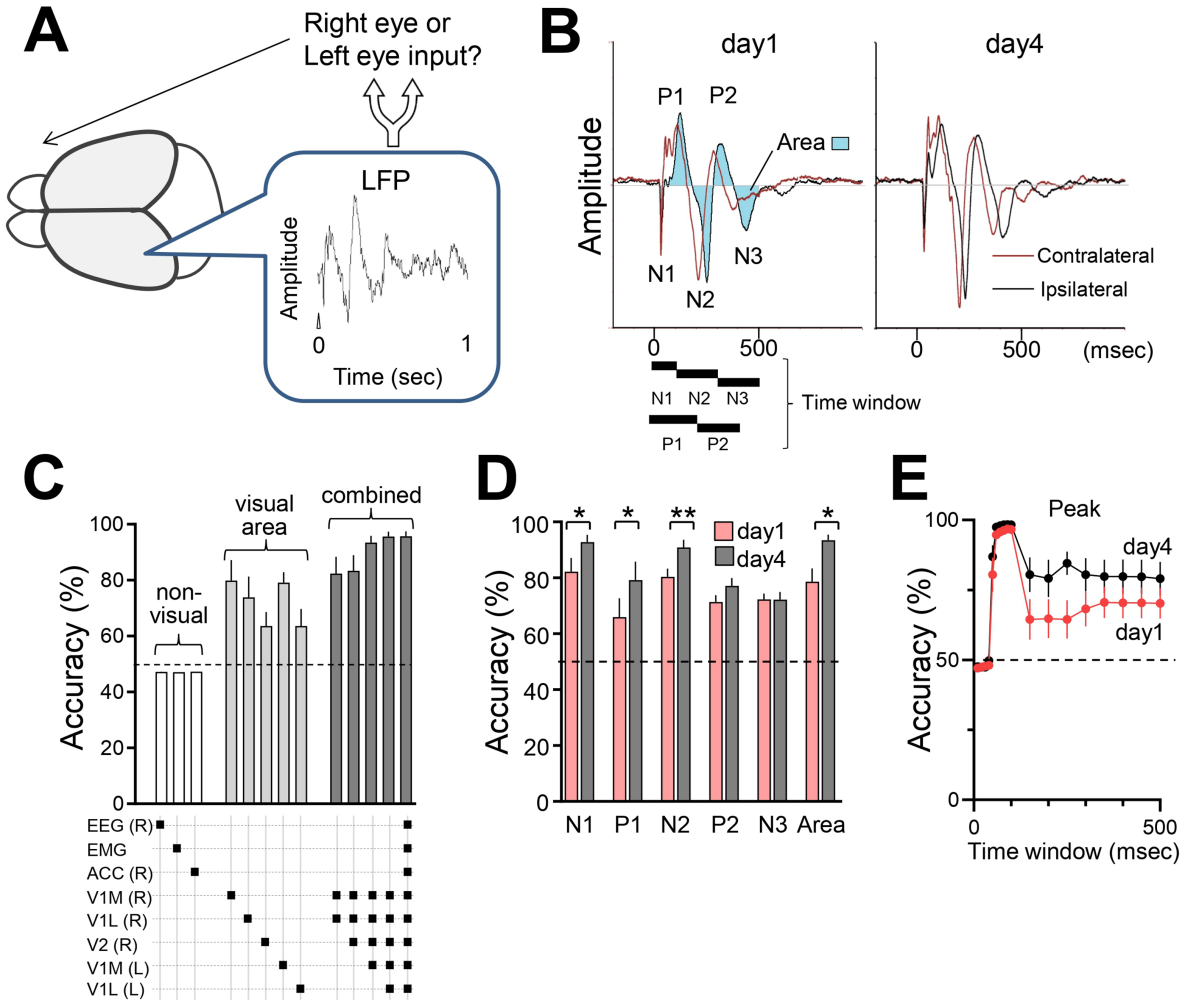
Decoding brain responses using a support vector machine (SVM) classifier **(A)** Estimation of eye input (right or left) from single brain responses using SVM. **(B)** VEP parameters (positive peak, negative peak, and area) used for the SVM classification. The time windows corresponding to each peak of VEPs were defined as follows: N1: 0–100 msec, N2: 100–300 msec, N3: 300–500 msec, P1: 0–200 msec, P2: 200–400 msec, and Area (shaded waveform area): 0–500 msec after stimulus onset (*t* = 0). **(C)** Classification accuracy using data from single or combinations of electrodes listed in the panel below. R: right hemisphere; L: left hemisphere. *N* = 6 mice. 50% is chance level. **(D)** Comparison of initial (300 pulses, day 1) and late sessions (300 pulses, day 4). *N* = 6 mice, Paired *t*-test, N1: *p = 0.0107, *t* = 3.965, df = 5; N2: **p = 0.0092, *t* = 4.118, df = 5; N3: *p* = 0.9825, *t* = 0.02304, df = 5; P1: *p = 0.0146, *t* = 3.661, df = 5; P2: P2 = 0.0760, *t* = 2.231, df = 5; Area: **p = 0.0091, *t* = 4.130, df = 5. Note that the simultaneously recorded visual responses of V1 (medial and lateral binocular regions, both hemispheres) and V2LM were used to train the SVM. **(E)** Accuracy using LFP waveform areas under the curve with different time windows ranging from 0 to 500 msec. Accuracies were calculated using information from all electrodes (6 mice).

Changes in brain responses would reflect the reorganized sensory information processing induced by sensory experience. To assess changes in sensory processing, we used a support vector machine (SVM) classifier to decode visual inputs (right or left eye) from VEP data (Figures 7A,B). Seventy-five percent of the visual response data from head-fixed mice were used to train the SVM classifier, while the remaining 25% of the data were reserved for testing the classifier’s performance. SVM analysis based on VEP peaks (P1, P2, N1, N2, N3) and the area under the curve revealed high classification accuracy, especially with visual cortex data compared with non-visual EEG or ACC data (Figure 7C). The classifier achieved over 95% accuracy with optimized time windows, with higher accuracy observed in responses from the late phase (day 4) compared with the initial phase (day 1) VEPs, suggesting enhanced sensory discrimination through the FVP (Figures 7D,E). No significant changes in VEP N1 amplitude were detected before and after FVP (Figures 2D–F). However, SVM analysis revealed a significant increase in discriminative power following FVP (Figure 7D). This is likely due to the SVM leveraging simultaneously recorded data from multiple visual regions, surpassing the simple averaging of the N1 amplitude (Figures 2D–F).

### N-methyl-d-aspartate (NMDA) receptor-dependent FVP

**Figure 8:**
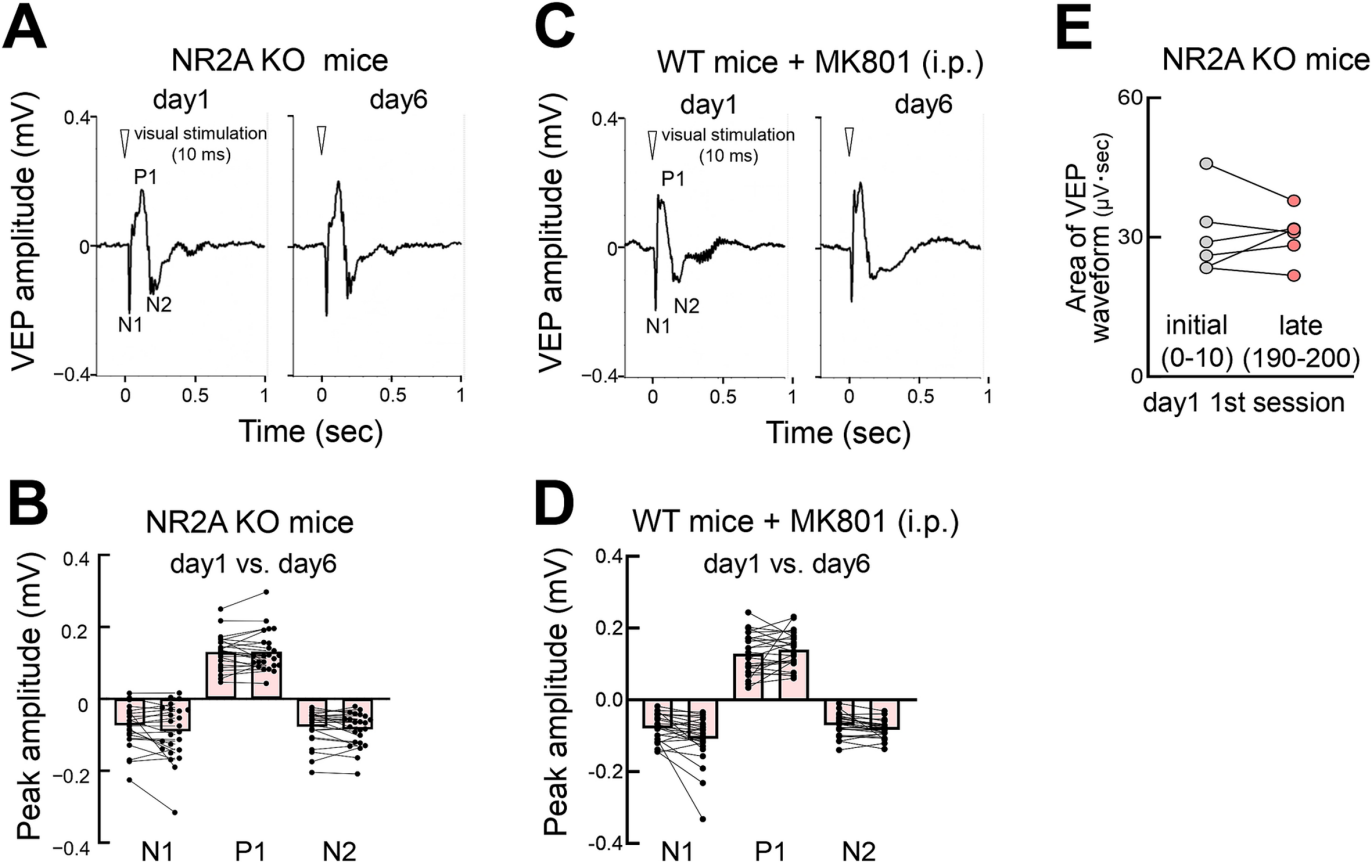
NMDA receptor-dependent FVP **(A)** VEPs in NR2A knockout mice on days 1 and 6 (contralateral eye stimulation). **(B)** Absence of changes in VEP peaks (day 1 vs. day 6) in NR2A knockout mice after visual stimulation (*N* = 6 mice, 12 recording sites, 24 recordings of contralateral or ipsilateral eye stimulation). Mann–Whitney test, N1: *p* = 0.5775; P1: *p* = 0.5844; N2: *p* = 0.4964. **(C)** VEPs of wild-type mice injected with MK801 intraperitoneally (0.5 mg/kg body weight) (day 1 vs. day 6, contralateral eye stimulation) 30 min before the start of visual stimulation. **(D)** Absence of FVP (day 1 vs. day 6) in MK801 treated wild type mice after visual stimulation. VEP peak N2 amplitudes (*N* = 6 mice, 12 recording sites, 24 recordings of contralateral or ipsilateral eye stimulation). Mann–Whitney test, N1: *p* = 0.1305; P1: *p* = 0.4552, N2: *p* = 0.1060. **(E)** Impaired rapid potentiation in NR2A KO mice. Visual response (area under waveform) during the initial stimulation (0–10 pulses) vs. late stimulation (190–200 pulses) on day 1. Paired *t*-test, *p* = 0.924, 6 mice, *t* = 0.1002, df = 5.

NMDA receptors play a crucial role in plasticity. We used *Grin2a* (a gene encoding NR2A) homozygous knockout (NR2A KO) mice (Sakimura et al., 1995; Fagiolini et al., 2003) to test the involvement of NMDA receptors in the FVP. NR2A protein, a subunit of NMDA receptors, develops postnatally and is incorporated into NMDA receptors (Quinlan et al., 1999). Lack of the NR2A subunit decreases NMDA receptor currents in the visual cortex (Miyamoto et al., 2003), resulting in the loss of plasticity of orientation selectivity (Fagiolini et al., 2003) and sensory experience-dependent slow-wave activity during non-REM sleep in developing mice (Miyamoto et al., 2003). We found that these mice lacked FVP, implicating NMDA receptor function in this plasticity process (Figures 8A,B), as well as other types of visual receptive field properties and learning behaviors (Frenkel et al., 2006). Heterozygous NR2A knockout mice showed intermediate FVP compared with wild-type and homozygous knockout mice (Supplementary Figures S5A,B). Additionally, pharmacological inhibition of NMDA receptors in wild-type mice using MK801, an open-channel NMDA receptor blocker, before stimulation abolished FVP, confirming its dependence on NMDA receptor signaling (Figures 8C,D). Rapid potentiation was also impaired in NR2A KO mice (Figure 8E).

### FVP in freely behaving mice

**Figure 9:**
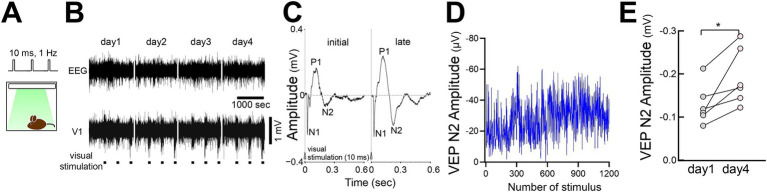
FVP in freely behaving mice **(A)** Visual stimulation (10 msec duration, 100 pulses at 1 Hz, 3 times of 100 pulses interleaved for 400 s per day) was applied to an awake behaving mouse from the ceiling of a recording chamber. **(B)** Representative LFP visual responses of a behaving mouse for consecutive 4 days. **(C)** VEP (day 1 vs. day 4) of a behaving mouse after visual stimulation. **(D)** Time course of the VEP N2 amplitude of a behaving mouse across visual stimulations (300 pulses/day for 4 days). **(E)** A significant increase in VEP N2 amplitudes (day 1 vs. day 4) in behaving mice after visual stimulation. *N* = 6 mice, paired *t*-test, *p = 0.0215, *t* = 3.299, df = 5.

We tested FVP in freely moving mice by delivering LED stimulation from above in a recording chamber (Figure 9A). VEP recordings showed that the visual response amplitudes (P1 and N2 peaks) gradually increased with repeated stimulation, similar to head-fixed mice (Figures 9B–D). N2 peak amplitude significantly increased by day 4 compared with that on day 1 (Figure 9E).

### Sleep changes associated with FVP

**Figure 10:**
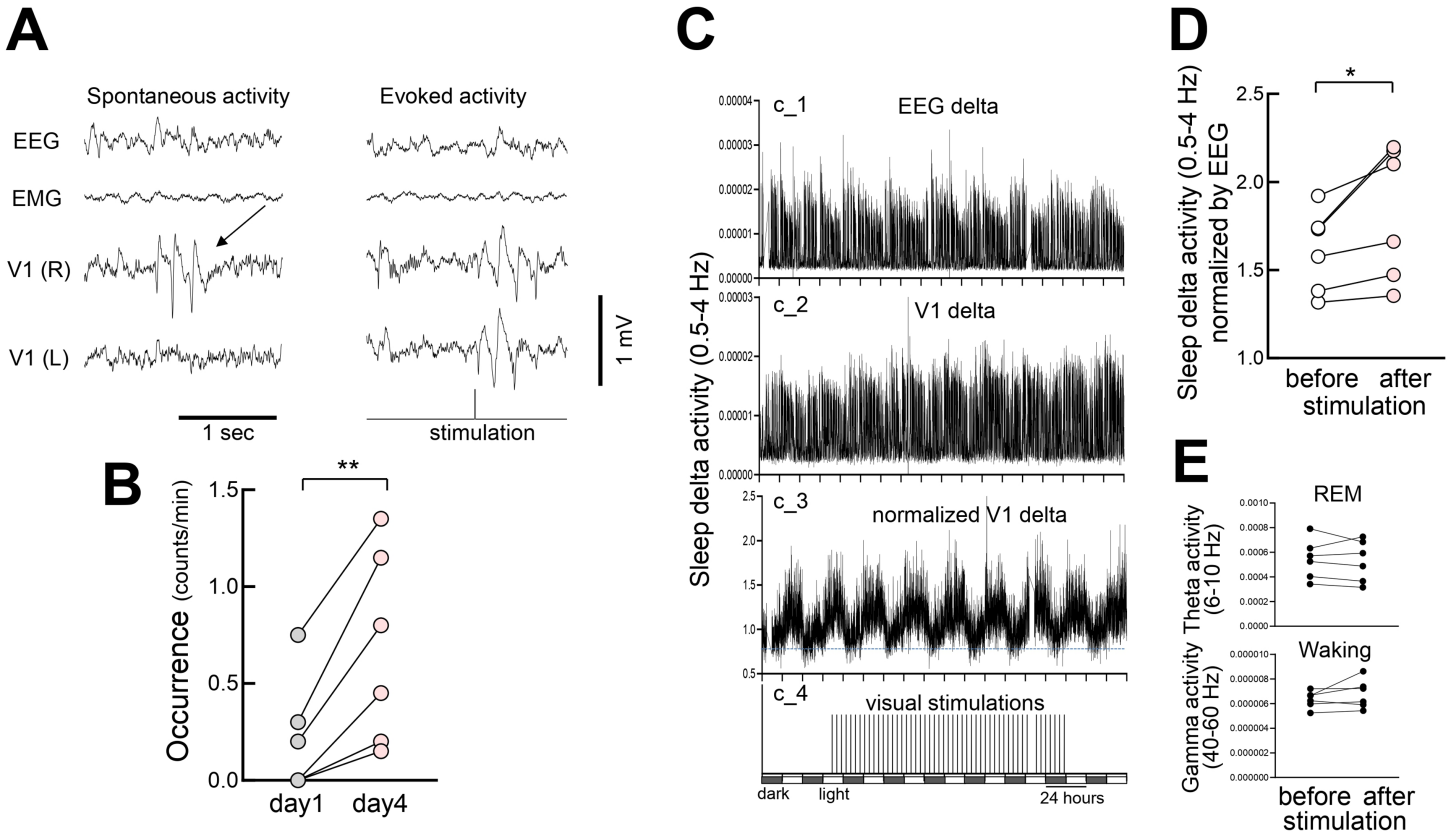
Increased slow-wave activity in V1 during non-REM sleep after FVP **(A)** (Left) Spontaneous 3–6 Hz spike-and-wave-like activity in V1 during waking. Spontaneous activity in V1 is sporadic and localized. (Right) Visually evoked activity in the same animal. **(B)** Increased frequency of spontaneous activity (day1 vs. day 4) after visual stimulation. *N* = 6 head-fixed mice, paired *t*-test, **p = 0.0072, *t* = 4.374, df = 5. **(C)** Time course of slow-wave activity (delta power, 0.5–4 Hz, 60 s bin) in a behaving mouse before, during, and after visual stimulation sessions. EEG and LFP delta activity did not include data when visual stimulation was provided and data during waking. Chronic recording was performed under light and dark (12:12) cycles over 8 days. (c_1) Somatosensory EEG delta activity. (c_2) V1 LFP delta activity concurrently recorded with EEG. (c_3) Normalized V1 LFP delta activity (divided by the EEG delta activity). Dotted horizontal line for visual inspection. (c_4) Visual stimulation. Each vertical bar indicates 300 pulse stimulations (10 msec duration, 1 Hz) that were interleaved for 3 h. **(D)** A significant increase in delta activity during sleep after visual stimulation (baseline before visual stimulations vs. days 4–10 after visual stimulation). *N* = 6 behaving mice, paired *t*-test, *p = 0.0374, *t* = 2.813, df = 5. **(E)** (Top) Theta activity (6–10 Hz) during REM sleep in the same animals. *N* = 6 behaving mice, paired *t*-test, *p* = 0.5898, *t* = 0.5756, df = 5. (Bottom) Gamma activity (40–60 Hz) during waking in the same animals, 6 behaving mice, paired t-test, *p* = 0.2327, *t* = 1.358, df = 5.

Occasional spontaneous 3–6 Hz oscillations have been observed in the visual cortex during wakefulness, displaying spike-and-wave patterns that resemble evoked responses (Senzai et al., 2019) and spontaneous activities (Miyamoto et al., 2019) (Figure 10A). These spontaneous oscillations became more evident after prolonged stimulation (day 4 vs. day 1) (Figure 10B). We also examined the effects of FVP on the activity during non-REM (NREM) sleep. Following multiple days (4–10 days, average 6.8 days) of stimulation (300 pulses at 1 Hz every 3 h), delta (0.5–4 Hz) rhythms in V1 increased significantly during NREM sleep, while somatosensory EEG activity remained unchanged (Figure 10C). Normalized V1 delta activity to EEG to reduce potential changes associated with global brain activity confirmed this increase (Figure 10D), whereas theta activity (6–10 Hz) during REM sleep or gamma activity during waking (40–60 Hz) remained stable (Figure 10E). This suggests that FVP selectively enhances delta activity during NREM sleep in V1.

## Discussion

In this study, we demonstrated that repeated flashing light stimulation potentiated visual responses in the adult mouse cortex. FVP has two distinct temporal processes: a rapid increase occurring within seconds to minutes, and a slower cumulative increase over hours to days. This form of visual plasticity is dependent on NMDA receptor function and is associated with increased slow-wave activity in the visual cortex during NREM sleep. Thus, the FVP may provide a simplified model for learning, memory, and neuropsychiatric disorders.

**Figure 11:**
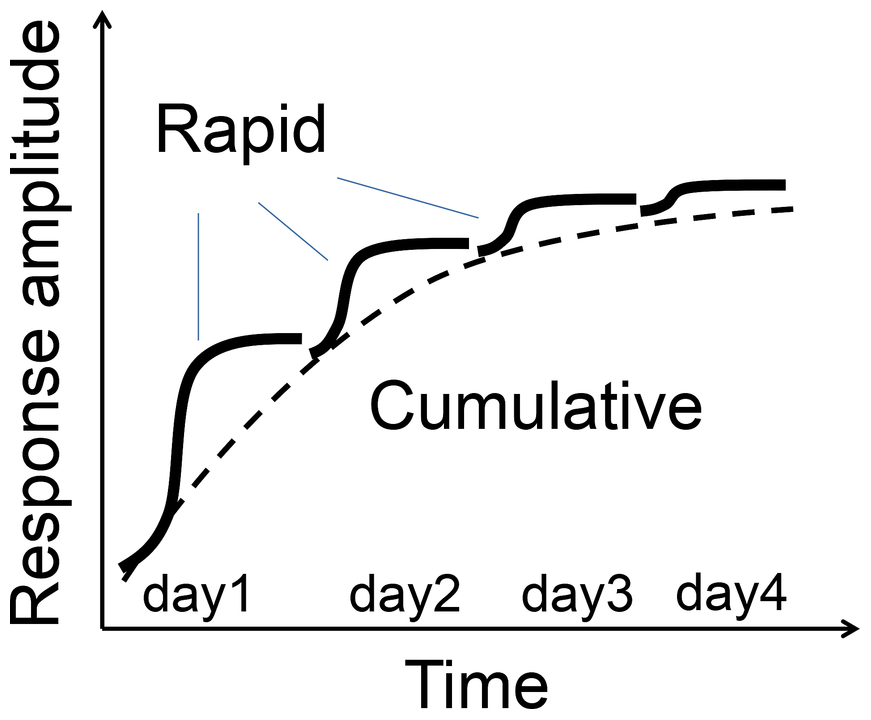
Two-process model of FVP schematic illustration of rapid and cumulative phases of FVP.

SRP and FVP are two forms of visual plasticity that exhibit distinct temporal characteristics and neural mechanisms. SRP, induced by repetitive exposure to specific grating orientations, emerges hours after stimulation and requires sleep for consolidation (Frenkel et al., 2006; Durkin and Aton, 2016; Montgomery et al., 2022). In contrast, FVP begins almost immediately during repetitive flash stimulation, with rapid potentiation followed by gradual increases in response amplitude over several days (Figure 11). SRP is marked by increases in early VEP peaks (N1, P1), whereas FVP shows enhancement primarily in later peaks (N2, P2, N3) without changes in N1, suggesting that FVP involves intracortical and cortico-subcortical processing rather than thalamocortical feedforward inputs (Schroeder et al., 1998). These differences highlight distinct neural pathways engaged by structured (gratings) versus unstructured (flashing) stimuli, with SRP and FVP likely engaging different circuits within the visual cortex while retaining common feedforward pathways across the cortical layers.

Flash stimulation, characterized by its brief duration and spatial uniformity, likely engages broader cortical circuits, including feedback and higher-order thalamic interactions. The longer latencies of VEP components associated with FVP (e.g., N2, N3) suggest involvement of cortico-cortical or cortico-subcortical interactions, potentially modulated by top-down cognitive processes (Heynen and Bear, 2001; Claar et al., 2023). NMDA receptor-dependent mechanisms, including long-term potentiation, are implicated in FVP, with hierarchical summation of synaptic inputs from V1 to higher-order visual areas or mediation by thalamic nuclei such as the lateral posterior nucleus (Murakami et al., 2022). Additionally, parvalbumin-positive fast-spiking interneurons, which are implicated in SRP and express NR2A, may contribute to FVP regulation (Kaplan et al., 2016).

Experience-dependent plasticity, such as SRP, and tasks involving sensory learning increase delta activity during NREM sleep in both humans and animals, aligning with our findings of enhanced slow-wave activity in V1 following FVP (Vyazovskiy et al., 2000; Huber et al., 2004). The observed increase in 3–6 Hz spontaneous oscillations suggests a link between FVP and NREM sleep oscillations. These results are consistent with evidence that thalamocortical circuits, which process sensory information during wakefulness, contribute to spontaneous oscillatory activity during sleep (Hill and Tononi, 2005; Durkin and Aton, 2019). Because local thalamic interactions regulate NREM sleep delta oscillations (Fernandez et al., 2018), FVP-induced changes may stem from cortico-thalamic interactions that affect sleep activity and, reciprocally, the consolidation of visual plasticity.

The two temporal phases of FVP—rapid potentiation and gradual enhancement—bear similarities to other learning processes such as cerebellar motor learning (Okamoto et al., 2011) and avian song learning (Deregnaucourt et al., 2005). In adaptive behavior, rapid learning followed by slower stabilization allows organisms to optimally adjust to dynamic environments. The decoding accuracy of visual responses using an SVM classifier also improved after the visual experience, suggesting that FVP enhances the brain’s discriminative capacity for sensory inputs. As a simplified and consistent model, the visual system offers an ideal framework for investigating memory processes because of its well-mapped thalamocortical circuits, which underpin sleep generation. Furthermore, the enhancement of the VEP amplitude through visual stimulation has been shown to correlate with improvements in visual detection ability (Clapp et al., 2012), suggesting that the visual plasticity observed in our experiments may represent a neural substrate of visual perceptual learning. This allows for the direct exploration of the interplay between sensory plasticity and sleep-dependent consolidation, further supporting its utility for translational research in learning and memory (Amedi et al., 2003; Beste et al., 2011; Lantz and Quinlan, 2021).

Similar to other forms of experience-dependent plasticity, the FVP depends on NMDA receptor activity. This was confirmed by the absence of FVP in Grin2a (NR2A)-knockout mice, consistent with findings in SRP and other plasticity models where NMDA receptor function is essential (Frenkel et al., 2006). In the visual system, NR2A deficiency leads to reduced ocular dominance plasticity, impaired orientation selectivity (Fagiolini et al., 2003), abnormal sleep spindles, and behavioral phenotypes, which are linked to schizophrenia-related phenotypes (Nelson and Valakh, 2015; Herzog et al., 2023). As a high-risk gene for schizophrenia, GRIN2A mutations are associated with NMDA receptor hypofunction, a key feature of the pathophysiology of the disorder (Singh et al., 2022). In NR2A KO mice, rapid potentiation was also inhibited, suggesting that rapid potentiation may be a prerequisite for the long-term cumulative process observed in wild-type mice. Furthermore, a lack of cortical plasticity might be relevant to certain symptoms in patients with schizophrenia. Given that VEP plasticity deficits have been observed in patients with schizophrenia (Cavus et al., 2012; Valstad et al., 2021), bipolar disorder (Valstad et al., 2021), major depression (Normann et al., 2007), and autism spectrum disorders (Ellis et al., 2021), the FVP may serve as a biomarker for tracking disease progression and treatment response.

SRP has provided significant insights into visual plasticity through the use of structured stimuli, such as orientation-specific gratings. Building on these findings, our study introduced FVP as a complementary approach (Supplementary Table S1). The spatially uniform nature of the light stimulation in FVP is expected to decrease complexity by avoiding higher-order visual information processing, such as orientation selectivity. We also employ relatively short 10 msec stimuli. This approach is thought to help reduce the temporal effects and complexity of visual stimulation on the response. Furthermore, in terms of temporally fluctuating light and dark stimuli, our method is considered to have a higher similarity to the visual stimuli that animals are likely to encounter in their natural environments, making them broadly applicable in translational and clinical research contexts. The ability to individually stimulate each eye and precisely control temporal patterns in visual stimuli facilitates spatial and temporal specific analyses. The versatility and simplicity of FVP make it well suited for various experimental paradigms to examine the interactions between visual plasticity and sleep, and the localized nature of the early visual system provides a useful model for in-depth mechanistic and computational investigations of visual plasticity (Toyoizumi et al., 2013).

## Data Availability

The original contributions presented in the study are included in the article/Supplementary material, further inquiries can be directed to the corresponding authors.

## Supplementary material

Supplementary Table S1 Comparison between SRP and FVP.

Supplementary Figure S1 Maintenance of rapid potentiation after visual stimulation sessions. **(A)** Reset Model: If the rapid potentiation disappears after the termination of visual stimulation, the magnitude of the visual response immediately before the end of the visual stimulation session should decrease compared to the initial response at the start of the next visual session. **(B)** Sustained Model: If the rapid potentiation is maintained after the termination of visual stimulation, the magnitude of the visual response immediately before the end of the visual stimulation session should be comparable to the initial response at the start of the next visual session. **(C)** The initial VEP N2 response at the start of the first session was normalized to 1. On day 1 and day 2, the following response ratios were measured and averaged across 14 mice: the ratio of the VEP amplitude immediately before the end of the first session to the first response at the start of the second session, the ratio of the VEP amplitude immediately before the end of the second session to the first response at the start of the third session, and the ratio of the VEP amplitude immediately before the end of the third session to the first response at the start of the first session on the following day. Data are presented as mean ± SEM.

Supplementary Figure S2 FVP in young mice (P30). VEP peak N2 amplitudes (4 mice, 8 recording sites, 16 recordings of contralateral or ipsilateral eye stimulation). Mann-Whitney test, N1: *p* = 0.2240; P1: 0.3414; N2: **p* = 0.0426.

Supplementary Figure S3 Brain states associated with FVP. Fluctuations in beta and gamma activity in the somatosensory cortex EEG, ACC LFP, and visual cortex V1L LFP associated with visual stimulation. Left column: beta activity in the 20–30 Hz band before, during (indicated by green bars), and after the visual stimulation session. Each visual stimulation session consisted of 200 stimuli. The data represent average of 6 mice with a bin size of 0.1 s. Right column: gamma activity in the 30–100 Hz band before, during, and after the visual stimulation session. Each visual stimulation session consisted of 200 stimuli. Beta and gamma activities were derived from the same recording data. Additionally, the EEG, ACC LFP, and V1L LFP activities were recorded simultaneously.

Supplementary Figure S4 Persistence of FVP after ACC inactivation. **(A)** Local application of muscimol to the anterior cingulate cortex 15 min before recording (unilateral injection). **(B)** Inactivation of the anterior cingulate cortex did not affect the FVP. VEP N2 amplitude (2 mice, 8 recording sites) after vehicle injection, and 1 mM or 10 mM muscimol 0.3 μL injected.

Supplementary Figure S5 Comparison of N2 amplitude across various conditions. **(A)** Intermediate FVP in NR2A heterozygous mice. VEP peak N2 amplitudes (3 mice, 6 recording sites, 12 recordings of contralateral or ipsilateral eye stimulation). Mann-Whitney test, N1: *p* = 0.8428; P1: *p* = 0.8428; N2: *p* = 0.7987. **(B)** Changes in VEP N2 amplitudes in adult wild-type (WT, 8 mice, 30 recordings), young WT (4 mice, 16 recordings), NR2A homozygotes (6 mice, 24 recordings), NR2A heterozygotes (3 mice, 12 recordings), and MK801-treated mice (6 mice, 24 recordings). VEP N2 amplitudes of the last day subtracted from those of the first day are plotted. One-way ANOVA, ****p* < 0.0001, Dunnett's multiple comparisons test, adult WT vs. Young, *p* = 0.8912; WT vs. MK801, ****p* = 0.0005; WT vs. NR2A homozygotes, ****p* = 0.0001; WT vs. NR2A heterozygotes, *p* = 0.0969.
