# Supplementary figures and images for: Rapid and cumulative adult plasticity in the mouse visual cortex
Source: Front Neural Circuits. 2025 Feb 28;19:1537305. doi: 10.3389/fncir.2025.1537305 (PMC11906431; doi:10.3389/fncir.2025.1537305)

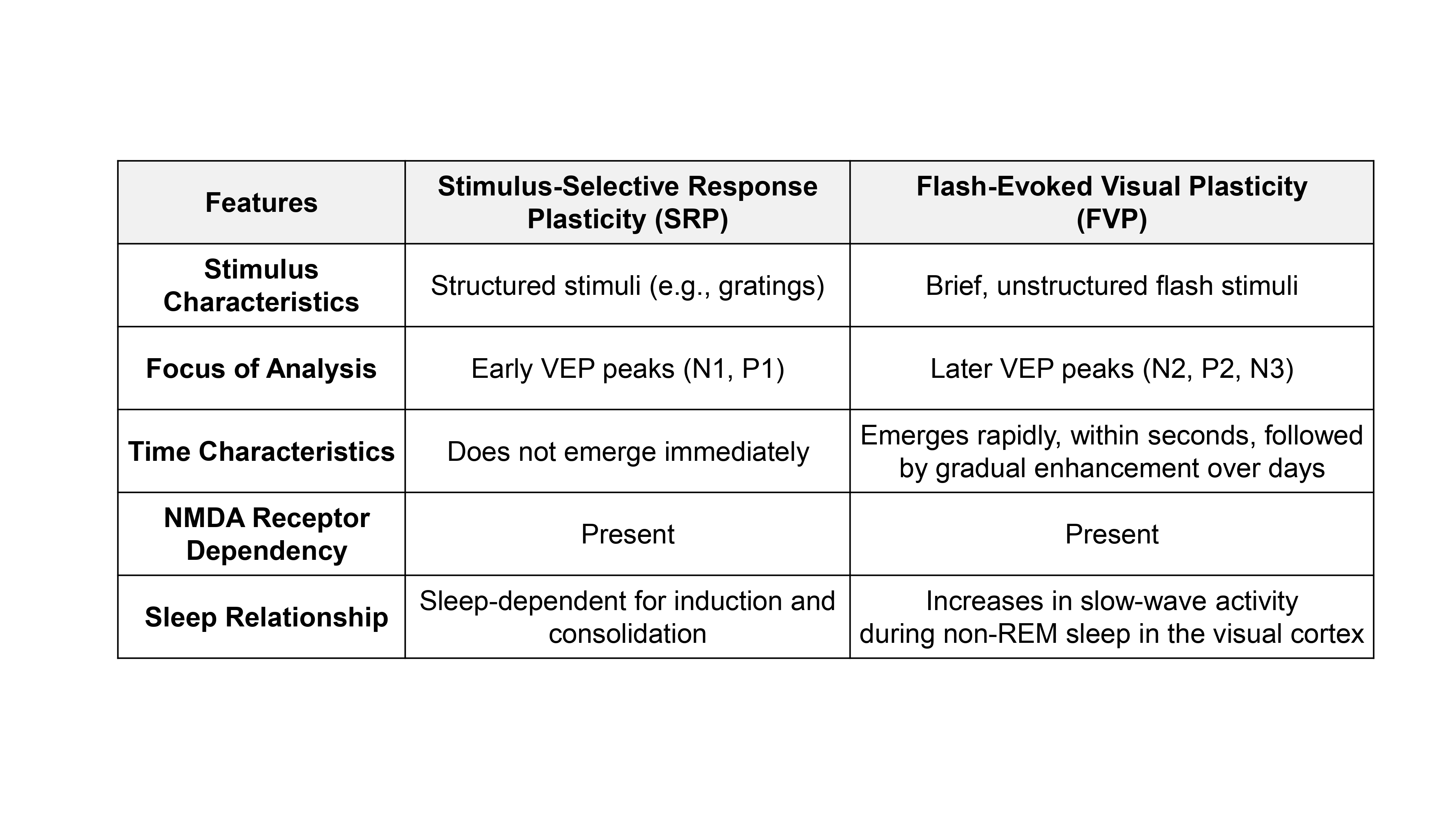

Supplement: Supplementary Table S1 — Comparison between SRP and FVP. [file Supplementary_file_1.jpg]

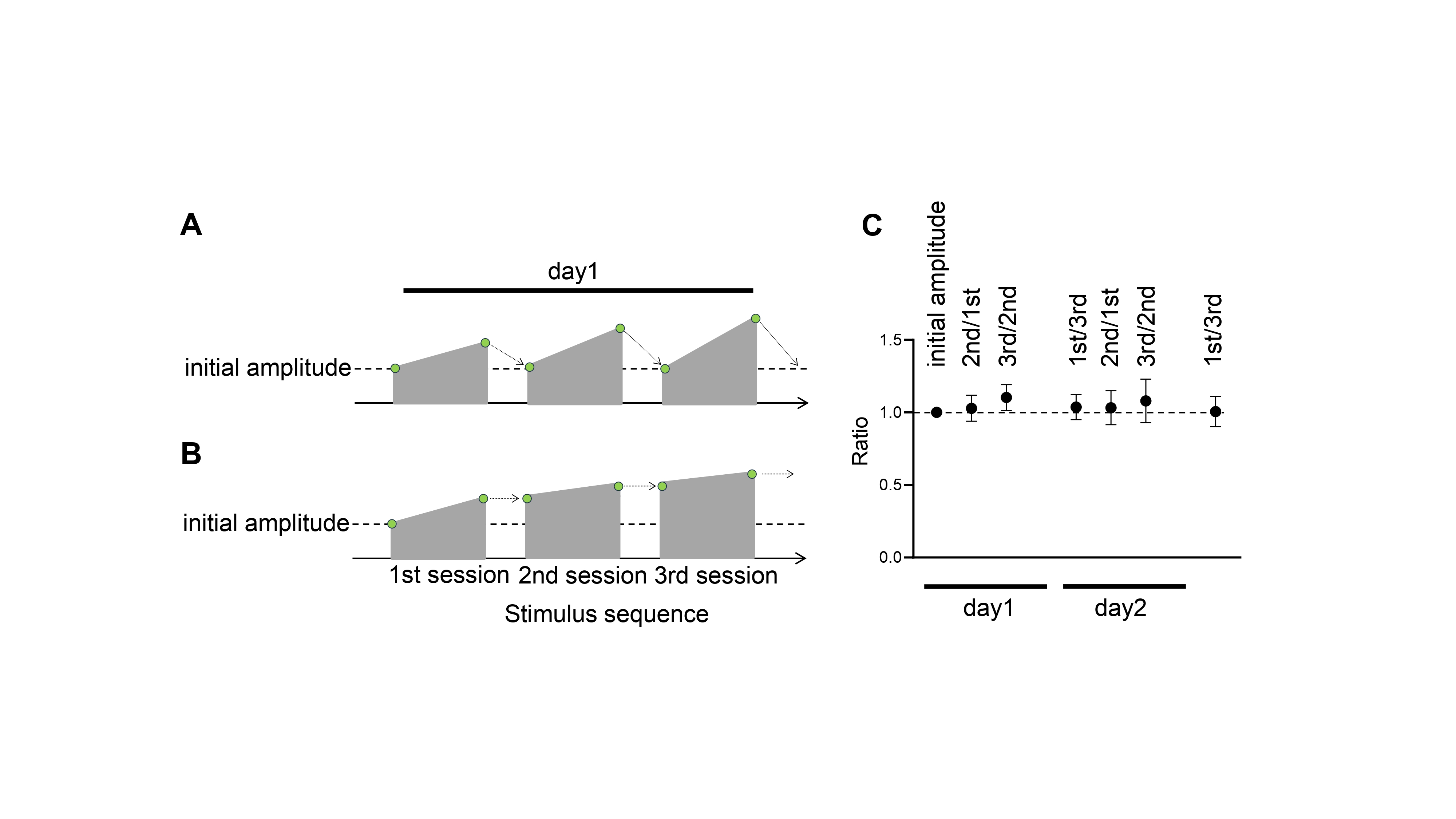

Supplement: Supplementary Figure S1 — Maintenance of rapid potentiation after visual stimulation sessions. (A) Reset Model: If the rapid potentiation disappears after the termination of visual stimulation, the magnitude of the visual response immediately before the end of the visual stimulation session should decrease compared to the initial response at the start of the next visual session. (B) Sustained Model: If the rapid potentiation is maintained after the termination of visual stimulation, the magnitude of the visual response immediately before the end of the visual stimulation session should be comparable to the initial response at the start of the next visual session. (C) The initial VEP N2 response at the start of the first session was normalized to 1. On day 1 and day 2, the following response ratios were measured and averaged across 14 mice: the ratio of the VEP amplitude immediately before the end of the first session to the first response at the start of the second session, the ratio of the VEP amplitude immediately before the end of the second session to the first response at the start of the third session, and the ratio of the VEP amplitude immediately before the end of the third session to the first response at the start of the first session on the following day. Data are presented as mean ± SEM. [file Image_1.jpg]

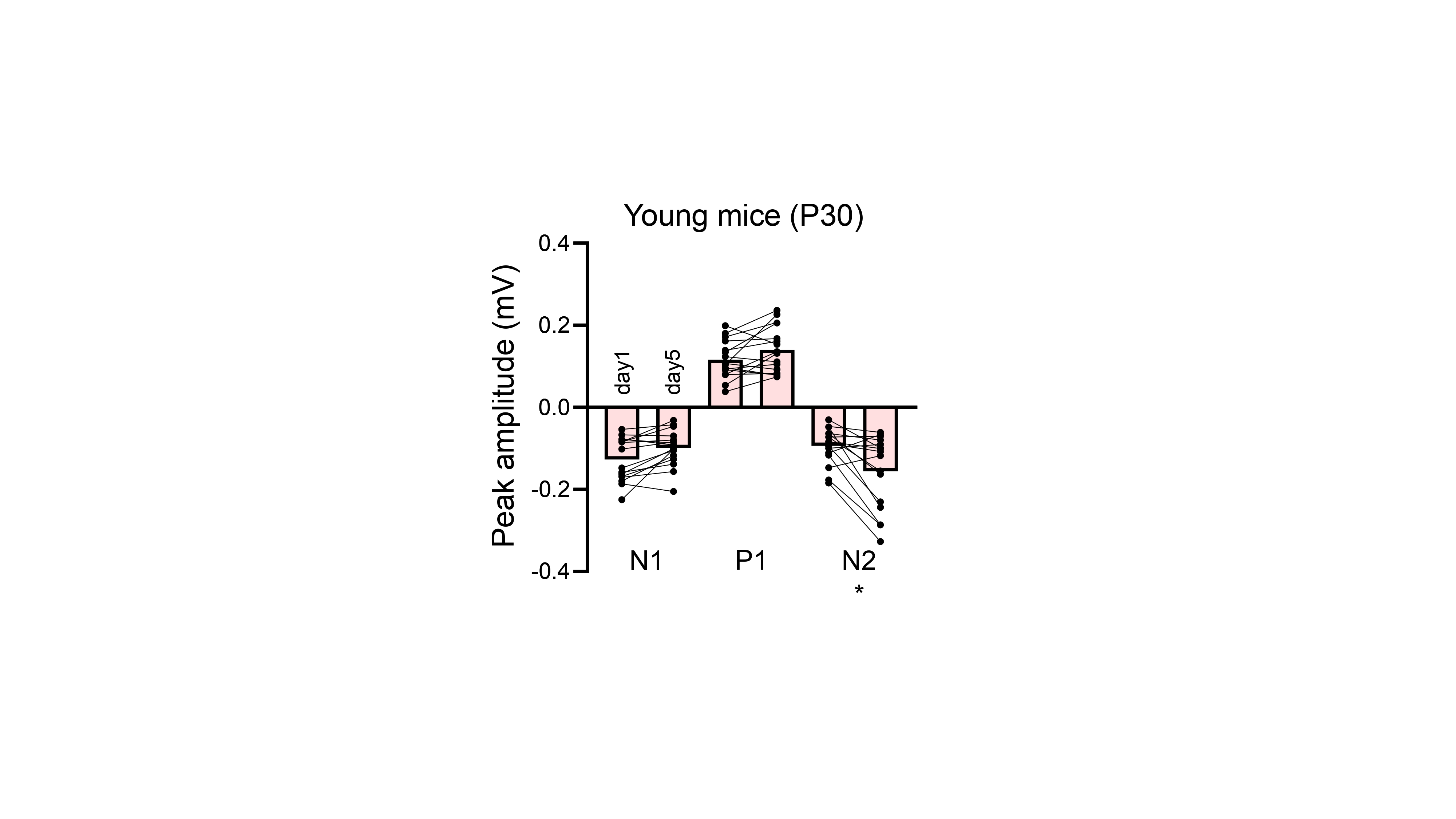

Supplement: Supplementary Figure S2 — FVP in young mice (P30). VEP peak N2 amplitudes (4 mice, 8 recording sites, 16 recordings of contralateral or ipsilateral eye stimulation). Mann-Whitney test, N1: p = 0.2240; P1: 0.3414; N2: *p = 0.0426. [file Image_2.JPEG]

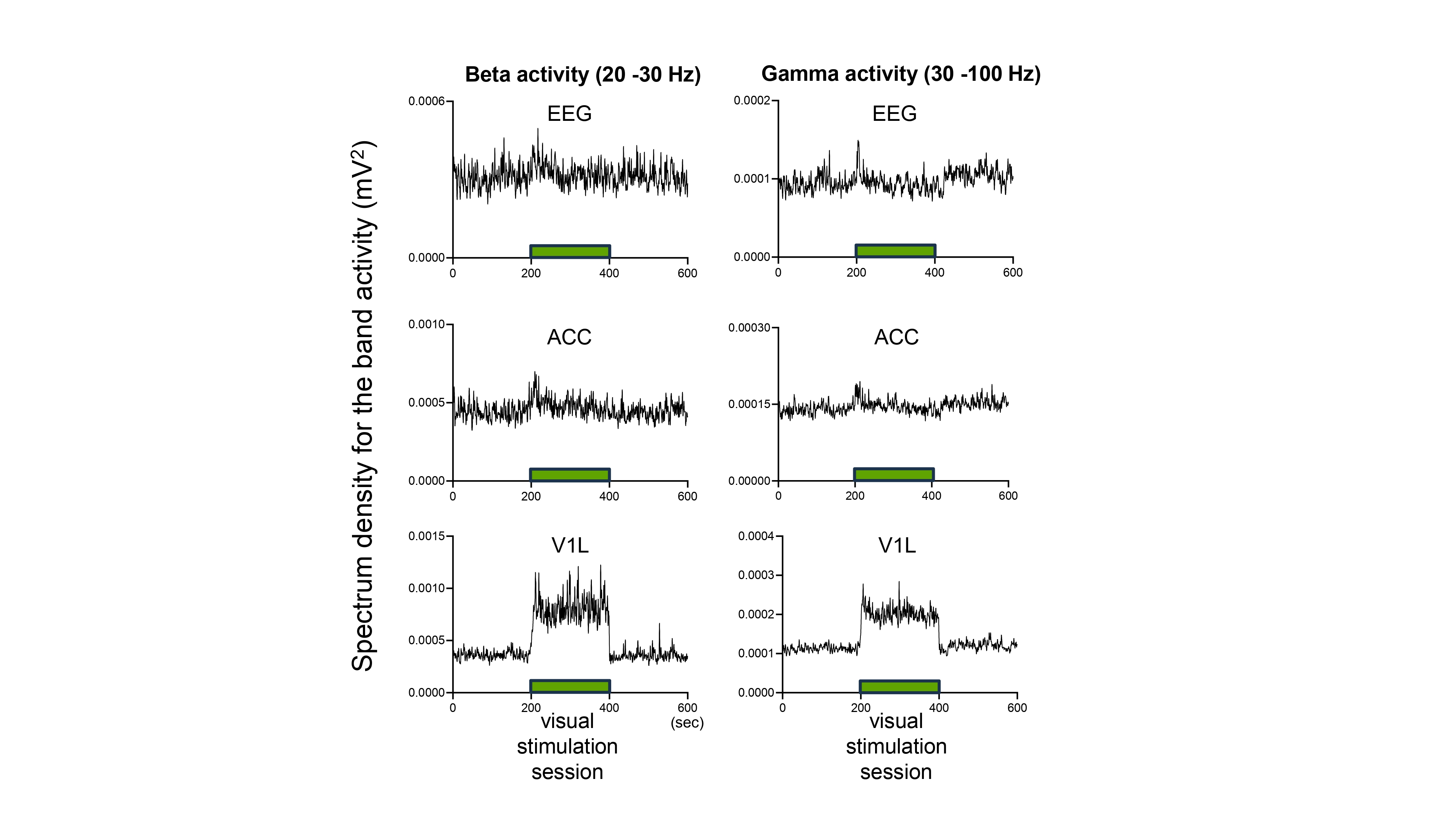

Supplement: Supplementary Figure S3 — Brain states associated with FVP. Fluctuations in beta and gamma activity in the somatosensory cortex EEG, ACC LFP, and visual cortex V1L LFP associated with visual stimulation. Left column: beta activity in the 20–30 Hz band before, during (indicated by green bars), and after the visual stimulation session. Each visual stimulation session consisted of 200 stimuli. The data represent average of 6 mice with a bin size of 0.1 s. Right column: gamma activity in the 30–100 Hz band before, during, and after the visual stimulation session. Each visual stimulation session consisted of 200 stimuli. Beta and gamma activities were derived from the same recording data. Additionally, the EEG, ACC LFP, and V1L LFP activities were recorded simultaneously. [file Image_3.JPEG]

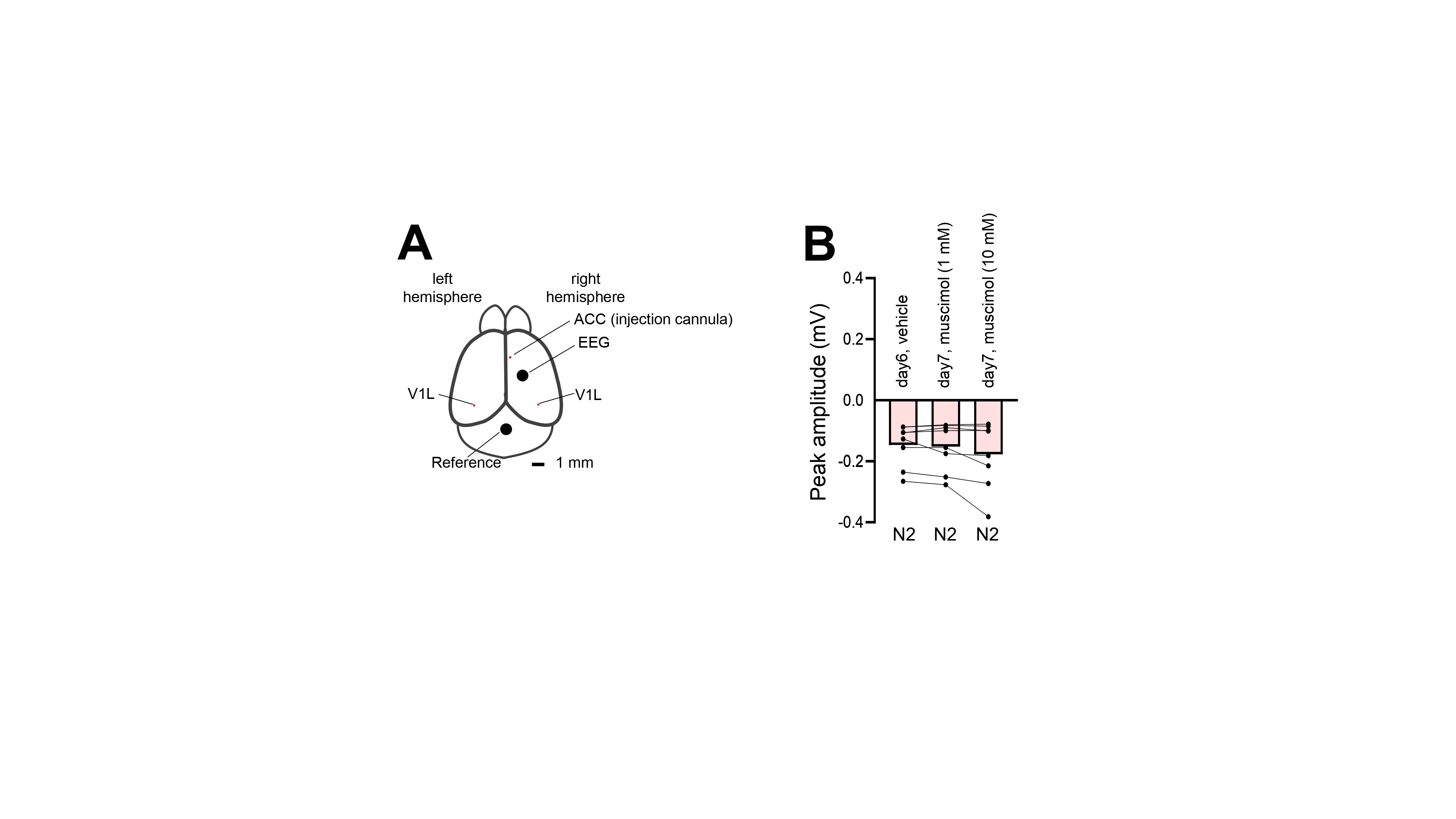

Supplement: Supplementary Figure S4 — Persistence of FVP after ACC inactivation. (A) Local application of muscimol to the anterior cingulate cortex 15 min before recording (unilateral injection). (B) Inactivation of the anterior cingulate cortex did not affect the FVP. VEP N2 amplitude (2 mice, 8 recording sites) after vehicle injection, and 1 mM or 10 mM muscimol 0.3 μL injected. [file Image_4.JPEG]

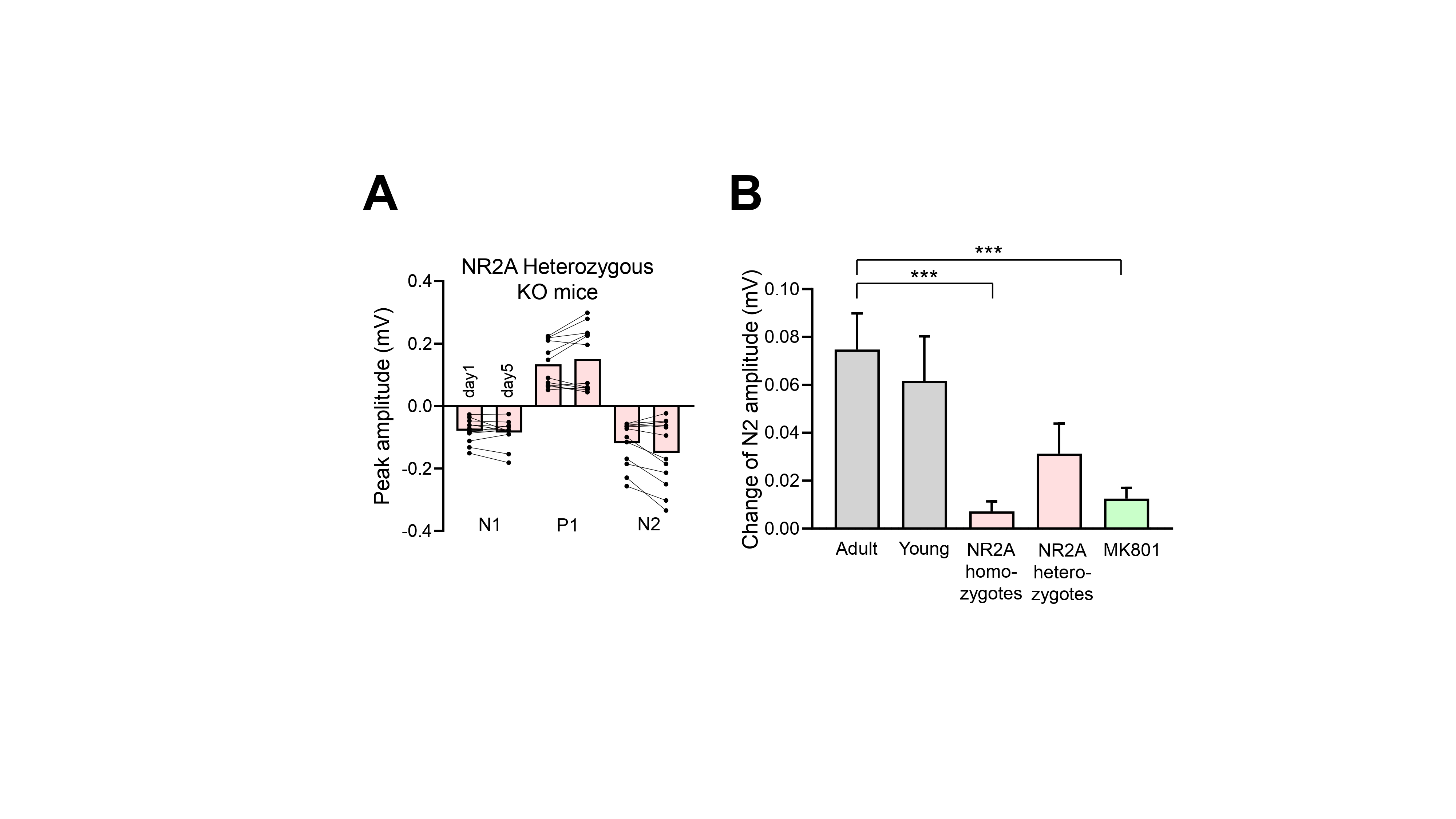

Supplement: Supplementary Figure S5 — Comparison of N2 amplitude across various conditions. (A) Intermediate FVP in NR2A heterozygous mice. VEP peak N2 amplitudes (3 mice, 6 recording sites, 12 recordings of contralateral or ipsilateral eye stimulation). Mann-Whitney test, N1: p = 0.8428; P1: p = 0.8428; N2: p = 0.7987. (B) Changes in VEP N2 amplitudes in adult wild-type (WT, 8 mice, 30 recordings), young WT (4 mice, 16 recordings), NR2A homozygotes (6 mice, 24 recordings), NR2A heterozygotes (3 mice, 12 recordings), and MK801-treated mice (6 mice, 24 recordings). VEP N2 amplitudes of the last day subtracted from those of the first day are plotted. One-way ANOVA, ***p < 0.0001, Dunnett's multiple comparisons test, adult WT vs. Young, p = 0.8912; WT vs. MK801, ***p = 0.0005; WT vs. NR2A homozygotes, ***p = 0.0001; WT vs. NR2A heterozygotes, p = 0.0969. [file Image_5.JPEG]
